# Mediterranean versus Western diet effects on cerebral cortical thickness and volume in cynomolgus monkeys

**DOI:** 10.1093/geroni/igaa057.1583

**Published:** 2020-12-16

**Authors:** Brett Frye, Suzanne Craft, Thomas Register, Jeongchul Kim, Christopher Whitlow, Samuel Lockhart, Carol Shively

**Affiliations:** 1 Wake Forest School of Medicine, Winston-Salem, North Carolina, United States; 2 Wake Forest University Alzheimer’s Disease Research Center, Winston-Salem, North Carolina, United States

## Abstract

Diet may influence the risk for cognitive decline and neurodegenerative disorders, including Alzheimer’s disease (AD), but these relationships are difficult to study in humans. Cynomolgus macaques (Macaca fascicularis) are appropriate models for investigations of diet effects on the brain because, like humans, they are omnivorous, have complex central nervous systems, are susceptible to diet-induced diseases, and accumulate amyloid and tauopathies with age. Using structural magnetic resonance imaging, we examined diet effects on brain anatomy by measuring thickness and volume of several areas relevant to AD in 38 middle-aged females, at baseline and after Mediterranean or Western diet consumption for 36 months (equivalent to a 9-year follow-up in humans). Using repeated measures analysis, cortical thicknesses generally increased in the Western diet group. Western diets also resulted in increases in total brain volume and cortical gray matter and decreases in cerebrospinal fluid, white matter, and deep gray matter (striatum and thalamus) (all p’s≤0.05). In contrast, thicknesses and volumes generally remained unchanged in animals consuming Mediterranean diets. Taken together, these findings demonstrate that Western diets induce widespread structural shifts which may increase risk of cognitive decline and neuropathology, whereas Mediterranean diets may exert a stabilizing influence on the brain. This study provides important insights about the significance of diet on brain structure and lays the groundwork for future investigations to uncover the molecular underpinnings of diet-induced changes in the brain. Mediterranean diet may protect against structural changes in brain that occur with age in those consuming a Western diet.

